# Peripheral Cytokine Levels Differ by HPV Status and Change Treatment-Dependently in Patients with Head and Neck Squamous Cell Carcinoma

**DOI:** 10.3390/ijms21175990

**Published:** 2020-08-20

**Authors:** Daphne Mytilineos, Jasmin Ezić, Adrian von Witzleben, Joannis Mytilineos, Ramin Lotfi, Daniel Fürst, Chrysanthi Tsamadou, Marie-Nicole Theodoraki, Angelika Oster, Gunnar Völkel, Hans A. Kestler, Cornelia Brunner, Patrick J. Schuler, Johannes Doescher, Thomas K. Hoffmann, Simon Laban

**Affiliations:** 1 Department of Otorhinolaryngology and Head & Neck Surgery, University Medical Center Ulm, 89070 Ulm, Germany; daphne.mytilineos@uniklinik-ulm.de (D.M.); Jasmin.Ezic@uniklinik-ulm.de (J.E.); a.von-witzleben@soton.ac.uk (A.v.W.); marie-nicole.theodoraki@uniklinik-ulm.de (M.-N.T.); osterangelika@web.de (A.O.); cornelia.brunner@uniklinik-ulm.de (C.B.); patrick.schuler@uniklinik-ulm.de (P.J.S.); johannes.doescher@uniklinik-ulm.de (J.D.); t.hoffmann@uniklinik-ulm.de (T.K.H.); 2 CRUK and NIHR Experimental Cancer Medicine Center & School of Cancer Sciences, Faculty of Medicine, University of Southampton, Southampton SO17 1BJ, UK; 3 Institute for Clinical Transfusion Medicine and Immune Genetics, German Red Cross Blood Transfusion Service, 89081 Ulm, Germany; joannis.mytilineos@zkrd.de (J.M.); r.lotfi@blutspende.de (R.L.); daniel.fuerst@uni-ulm.de (D.F.); c.tsamadou@blutspende.de (C.T.); 4 Institute for Transfusion Medicine, University Hospital Ulm, 89081 Ulm, Germany; 5 Institute for Medical Systems Biology, Ulm University, 89081 Ulm, Germany; gunnar.voelkel@googlemail.com (G.V.); hans.kestler@uni-ulm.de (H.A.K.)

**Keywords:** cytokines, head and neck cancer, human papillomavirus (HPV), longitudinal study, Fas, perforin, High-Mobility-Group-Protein B1(HMGB1), tumor immune evasion, inflammation

## Abstract

Cytokines and immune mediators play an important role in the communication between immune cells guiding their response to infectious diseases or cancer. In this study, a comprehensive longitudinal analysis of serum cytokines and immune mediators in head and neck squamous cell carcinoma (HNSCC) patients was performed. In a prospective, non-interventional, longitudinal study, blood samples from 22 HNSCC patients were taken at defined time points (TP) before, during, and every 3 months after completion of (chemo)radio)therapy (CRT/RT) until 12 months after treatment. Serum concentrations of 17 cytokines/immune mediators and High-Mobility-Group-Protein B1 (HMGB1) were measured by fluorescent bead array and ELISA. Concentrations of sFas were significantly elevated during and after CRT/RT, whereas perforin levels were significantly decreased after CRT/RT. Levels of MIP-1β and Granzyme B differed significantly during CRT/RT by HPV status. Increased HMGB1 levels were observed at recurrence, accompanied by high levels of IL-4 and IL-10. The sFas increase and simultaneous perforin decrease may indicate an impaired immune cell function during adjuvant radiotherapy. Increased levels of pro-inflammatory cytokines in HPV+ compared to HPV− patients seem to reflect the elevated immunogenicity of HPV-positive tumors. High levels of HMGB1 and anti-inflammatory cytokines at recurrence may be interpreted as a sign of immune evasion.

## 1. Introduction

Head and neck squamous cell carcinoma (HNSCC) is diagnosed in almost 900,000 cases worldwide resulting in approximately 450,000 cancer deaths per year [1]. The curative treatment of locoregionally advanced HNSCC according to international guidelines is either surgery followed by risk-adapted adjuvant (chemo)radiotherapy (RT) or definitive chemoradiotherapy (CRT) [2]. A subgroup of HNSCC, typically oropharyngeal squamous cell carcinoma, can be attributed to high-risk human papillomaviruses (HPV) [3,4]. HPV-16 is the virus type found most frequently in those tumors. In comparison to HPV-negative patients, patients with HPV-positive tumors have a better prognosis, regardless of the type of conventional curative treatment [5,6,7]. The abundance of tumor-infiltrating lymphocytes is independently associated with improved survival. However, in HPV-positive tumors these immune infiltrates are generally denser than in HPV-negative tumors indicating a “hot” tumor immune microenvironment [8,9,10,11,12]. Further, the presence of HPV-specific immune cells in the tumor microenvironment is associated with a good prognosis [13]. In summary, HPV-positive tumors are characterized by a pronounced immune response which also results in high expression of interferon-gamma (IFN-γ) associated gene expression, the so-called IFN-γ signature [14].

Published studies investigating cytokine expression in HNSCC were mostly focused on the differences between healthy controls and patients with HNSCC [15,16,17] or in vitro studies of cytokine expression by HNSCC cell lines [18,19]. We have previously analyzed the effects of photodynamic therapy on peripheral cytokine concentrations [20]. To date, only one study investigated the effects of curative conventional therapy [21]. In that study, the impact of HPV-status on the cytokine profiles was not assessed and the majority of patients were treated non-surgically.

Here we present a prospective, longitudinal immune monitoring study of peripheral cytokine levels and pro-apoptotic immune mediators—henceforth addressed as immune mediators—during curative treatment of HNSCC, with a special focus on HPV-associated differences.

## 2. Results

### 2.1. Patients

The study included 22 HNSCC patients prospectively observed within a longitudinal clinical study of curative conventional treatment. The American Joint Committee on Cancer (AJCC) cancer staging manual version 7 was used. Detailed patient characteristics are provided in Table 1. Among the 22 patients in this study, 14 patients were HPV negative and 7 patients with an oropharyngeal primary were HPV positive. For one patient HPV status was missing. In accordance with oncologic guidelines, 18 patients underwent surgery as first-line treatment. Three of those patients (17%) had no indication for adjuvant treatment and two (11%) declined adjuvant treatment. Eight patients received adjuvant RT (44%) and 5 patients adjuvant CRT (28%). Four patients were treated with primary CRT.

**Figure 1:**
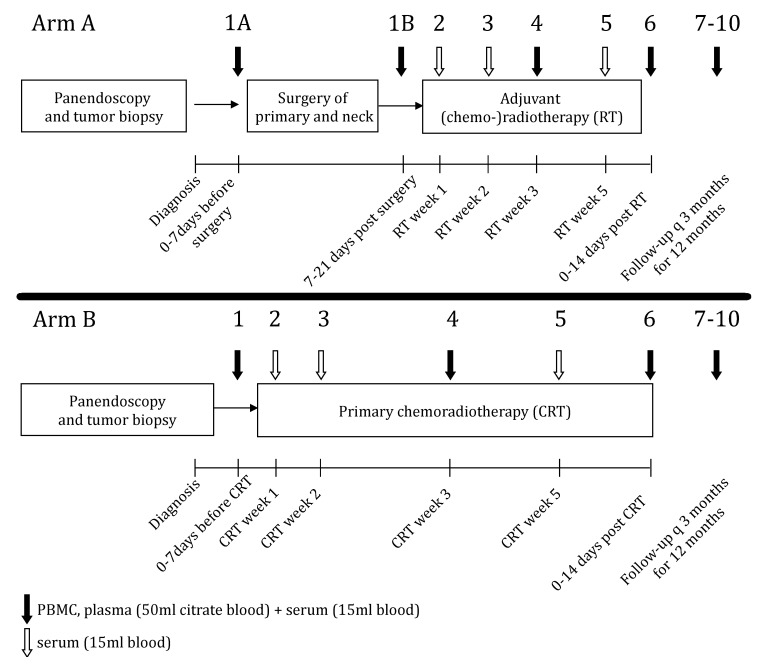
Patient sampling scheme. Patients were allocated to Arm A (surgical treatment + risk-adapted adjuvant radiotherapy, RT ± chemotherapy) or Arm B (chemoradiotherapy, CRT) after interdisciplinary tumor board assessment. Sampling was performed as defined per protocol at specified parallel time points in Arm A and B. Full arrows represent 50 mL whole blood samples (citrate-buffered) and 15 mL serum samples (serum tubes). Open arrows represent a 15 mL sample for serum only (serum tubes). Serum (cytokine and immune mediator panel) and plasma (HMGB1) samples were used in this study. The baseline sample (Arm A: Sample 1A, Arm B: Sample 1) was taken prior to treatment initiation. In Arm A, an additional sample was taken during the post-surgical recovery phase (sample 1B). Samples were also taken mid-RT/mid-CRT (sample 4) and at the end of RT/CRT (sample 6) as well as every 3 months during post-treatment follow-up for a total of 12 months after the end of treatment (sample 7–10).

The sample collection scheme for serum and plasma samples is depicted in Figure 1. Out of the 238 planned serum samples, 192 (81%) were available. Fifteen patients (68%) had 8 or more available samples. Reasons for missing samples were withdrawal from longitudinal sample collection during treatment (*n* = 3), treatment complications (*n* = 3), change of Tumor Node Metastasis classification (TNM) after surgical therapy voiding the need of adjuvant RT (*n* = 2), or decline of adjuvant treatment (*n* = 2) and an unreported prior malignancy (*n* = 1).

### 2.2. Altered Serum Perforin and sFas Levels during Treatment

**Figure 2:**
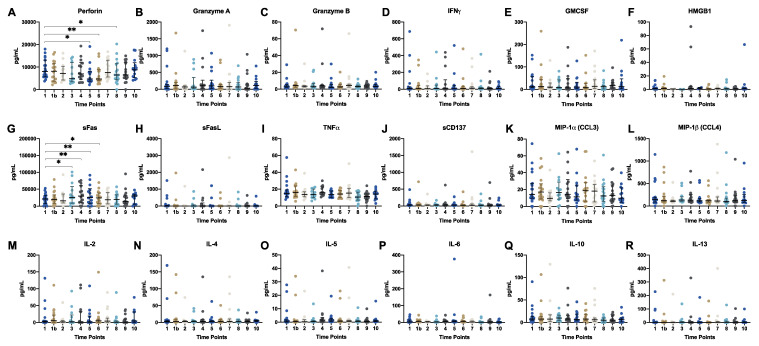
Serum cytokineand immune mediatorlevels over time: (**A**) Perforin, (**B**) Granzyme A, (**C**) Granzyme B, (**D**) IFN-γ, (**E**) GMCSF, (**F**) HMGB1, (**G**) sFas, (**H**) sFasL, (**I**) TNF-γ, (**J**) sCD137, (**K**) MIP-1α (CCL3), (**L**) MIP-1β (CCL4), (**M**) IL-2, (**N**) IL-4, (**O**) IL-5, (**P**) IL-6, (**Q**) IL-10, (**R**) IL-13. The measured levels are presented on the y-axis and the sampling time points on the x-axis. Available patient samples at each time point are shown in grouped scatter plots. Medians are presented as horizontal bars and the error indicators present the interquartile range. Significant differences are indicated by asterisks. *: *p* < 0.05, **: *p* < 0.01.

Serum levels of the pro-apoptotic immune mediators Perforin, Granzyme A, Granzyme B and the cytokines IFN-**γ**, GMCSF, soluble Fas receptor (sFas), soluble Fas ligand (sFasL), TNFα, sCD137, MIP-1α (CCL3), MIP-1β (CCL4), IL-2, IL-4, IL-5 IL-6, IL-10, and IL-13, as well as plasma levels of HMGB1 were measured in all patients at each available time point. The distribution of cytokine and immune mediator serum concentration for the measured time points is displayed in Figure 2. Overall, serum concentration of most cytokines and immune mediators did not change significantly during therapy and follow up. An exceptionally wide range and distribution of serum concentrations were observed for sFas and Perforin considering the whole observation period and the complete cohort. In contrast, for Granzyme B, IL-5, TNFα, MIP-1α (CCL3), HMGB1, IL-10, IL-1, and IL4, the interquartile range of all available samples during the study period was <200 pg/mL (Supplementary Figure S1).

Comparing related samples longitudinally, serum perforin levels showed a significant decrease at time point 5 (week five of adjuvant treatment; *p* = 0.023), time point 6 (after completion of adjuvant treatment; *p* = 0.002) and time point 8 (6 months after completion of adjuvant treatment; *p* = 0.028) when compared to the respective baseline concentration (Figure 2A). Furthermore, sFas serum concentration showed a significant increase at time point 3 (week two of adjuvant treatment; *p* = 0.023), time point 4 (week three of adjuvant treatment; *p* = 0.003), time point 5 (p = 0.008) and time point 6 (*p* = 0.019) compared to the respective baseline concentration (Figure 2G).

### 2.3. Increased Serum MIP-1β (CCL4) and Granzyme B in HPV-Positive Patients during Treatment

**Figure 3:**
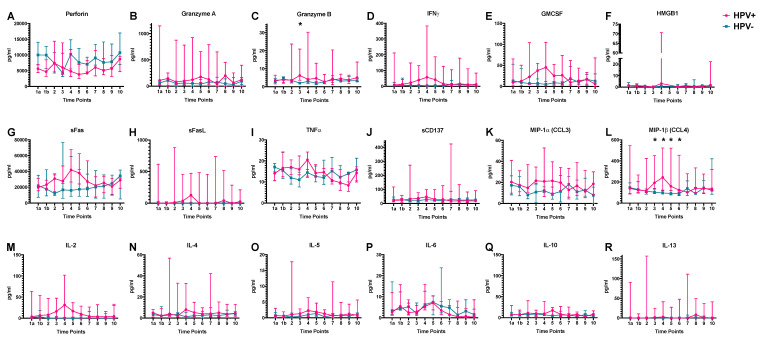
Serum cytokineand immune mediatorlevels in HPV-positive and HPV-negative patients: (**A**) Perforin, (**B**) Granzyme A, (**C**) Granzyme B, (**D**) IFN-γ, (**E**) GMCSF, (**F**) HMGB1, (**G**) sFas, (**H**) sFasL, (**I**) TNF-γ, (**J**) sCD137, (**K**) MIP-1α (CCL3), (**L**) MIP-1β (CCL4), (**M**) IL-2, (**N**) IL-4, (**O**) IL-5, (**P**) IL-6, (**Q**) IL-10, (**R**) IL-13. The measured levels are presented on the y-axis and the sampling time points on the x-axis. Available patient samples at each time point are shown as line graphs with median and interquartile range (magenta = HPV+, teal = HPV−). Significant differences are indicated by asterisks. *: *p* < 0.05.

To understand the effect of HPV status on the cytokine and immune mediator levels, these were measured at the defined time points between HPV-positive and HPV-negative HNSCC patients (Figure 3). Most of the cytokines, especially immune-stimulatory and pro-apoptotic agents, showed increased median serum levels in HPV-positive patients during adjuvant therapy when compared to HPV-negative patients. In particular, a significant increase was observed for MIP-1β serum concentration at time points 3, 4, 5, and 6 (*p* = 0.009, *p* = 0.007, *p* = 0.026, and *p* = 0.021, respectively), and a short-term increase for Granzyme B serum levels at time point 3 (*p* = 0.01).

Whereas median Perforin levels showed a tendency to lower values in HPV-positive patients, IFN-**γ**, GMCSF, sFas, sFasL, TNFα, MIP-1α (CCL3) and IL-2 tended to increased median serum levels in HPV-positive patients during radiotherapy. Notably, time point 4 in the middle of radiotherapy indicates clear peaks for cytokines such as IFN-**γ**, GMCSF, sFas, sFasL, IL-2, IL-4 which is in accordance with the most significant change of MIP-1β at TP4.

### 2.4. Stronger Cytokine and Immune Mediator Level Correlation in HPV-Positive than in HPV-Negative Patients

**Figure 4:**
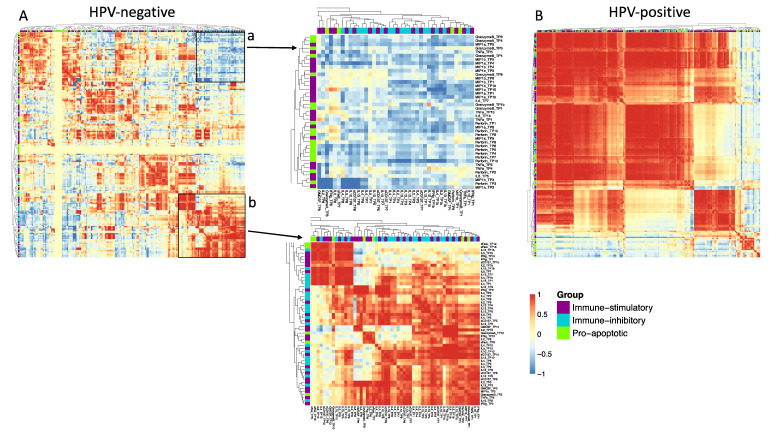
Correlation matrices of all cytokines and immune mediators at all time points. Patients were separated by HPV-status. Pearson correlation coefficients between all cytokines and time points were graphed and hierarchical clustering was performed using Euclidian distance. Red represents positive, and blue negative correlation. Cytokines were attributed to three groups: Purple = immune stimulatory cytokines, turquoise = immune inhibitory cytokines, green = pro-apoptotic immune mediators. (**A**) Correlation coefficients in HPV- patients. Two regions are highlighted: Region a with a cluster of negative correlation coefficients and Region b with a cluster of positive correlation coefficients. (**B**) Correlation coefficients in HPV+ patients.

To further evaluate differences in the cytokine and immune mediator profiles on a higher abstraction level, correlations of all cytokine and immune mediator concentrations at the different time points were performed. Patients were separated according to HPV-status, and Pearson correlation of all cytokine and immune mediator levels with each other at all time points were performed (Figure 4). The correlation matrices clearly show a stronger and more uniform correlation of the cytokine and immune mediator co-expression in the HPV-positive patients as compared to the HPV-negative patients. Except for the two marked clusters (Figure 4A,B), there are no clear patterns present in the HPV-negative patients. The upper right cluster (Figure 4A, a) shows that MIP-1α, MIP-1β, and perforin throughout the time points have a strong negative correlation with several IL-2 family members, mostly IL-4 and IL-13. In addition, the lower right cluster (Figure 4A, b) shows a strong, positive correlation within the members of the IL-2 family (IL-4, IL-5, IL-10, and IL-13) at multiple time points during the study. Interestingly, sFasL expression compared between different time points does not correlate at all. In the HPV-positive group, a uniform positive correlation is observed within most of the cytokines throughout the measured period, which is in line with the inflammatory phenotype described for HPV-positive patients.

Considering the whole cohort, the correlation matrix shows a fairly strong positive correlation between the variables, which seems to be driven by HPV-positive patients (Supplementary Figure S2).

**Figure 5:**
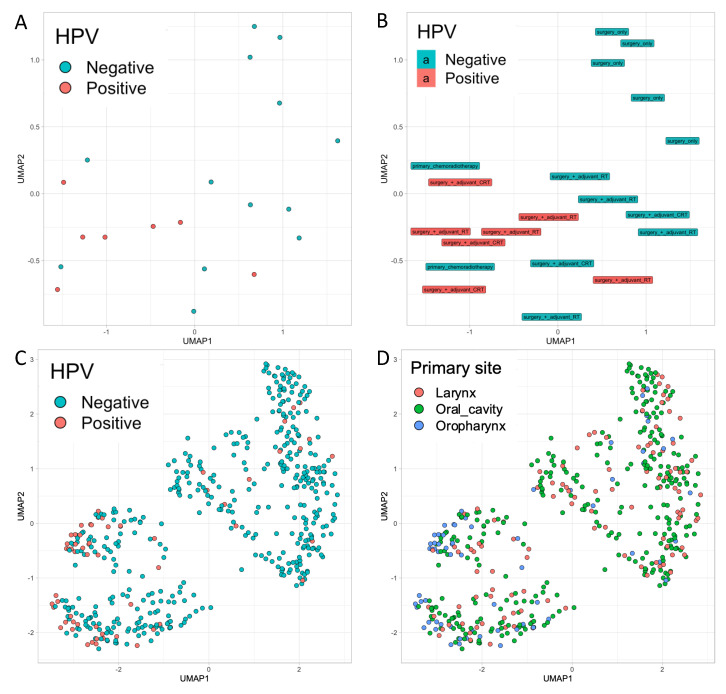
Uniform manifold approximation and projection (UMAP) clustering based upon cytokine and immune mediator levels. Each dot/box represents one patient. (**A**,**B**) UMAP clustering using all cytokines and immune mediators at the respective time points as variables for the IRECT cohort. C and D: UMAP clustering of cytokine and immune mediator RNA expression data from the TCGA cohort by (**C**) HPV-status and (**D**) primary site.

Because of the complex multidimensionality of the measured biomarkers at multiple time points, we performed Uniform Manifold Approximation and Projection (UMAP) clustering with each cytokine and immune mediator concentration at a given time point as a variable, to see which patient clusters would emerge (Figure 5). HPV status is strongly reflected in the clustering (Figure 5A). Additionally, primary therapy seems to be represented in the formed clusters (Figure 5B). Two HPV-negative patients received primary CRT cluster with the HPV-positive group. To confirm our findings of HPV-status as a major determinant of the cytokine and immune mediator profile, we investigated HNSCC samples from the TCGA cohort and used RNA-Seq data for the measured cytokine panel within primary tumor samples. UMAP clustering of TCGA data showed distinct clustering, which, as in our cohort, was primarily determined by HPV status (Figure 5C). However, among HPV-negative patients, a subgroup of highly inflamed tumors cluster with HPV-positive patients. Such inflamed tumors can be found in all primary sites (Figure 5D).

### 2.5. Increase in Plasma HMGB1 at Time point of Recurrence

To assess the influence of recurrent disease on serum levels of HMGB1 and inhibitory interleukins (IL-4, IL-10, IL-13) the individual courses of patients with recurrence were graphed (Supplementary Figure S3). We observed an increase of plasma HMGB1 at the time of recurrence or prior to the detection of recurrence in 4 of 5 patients with available samples. However, not every HMGB1 increase was associated with a recurrence indicating a low specificity. Moreover, the course of inhibitory cytokines in patients with recurrence showed either overall high IL-4 levels (at all time points) or an increase before recurrence (Supplementary Figure S3B).

## 3. Discussion

We performed a prospective, longitudinal monitoring study of peripheral cytokine and immune mediator levels during curative treatment of HNSCC. Cytokines play a variety of roles in the immune response depending on the immune-contexture. In addition to that, they can be used as biomarkers, especially in immunotherapy [22]. We observed significantly lower perforin levels and higher sFas levels during (C)RT. Most previous research was focused on comparing the cytokine levels of healthy controls and tumor patients [15,16,17] and measuring cytokine release in vitro [18,19]. There is only one other study prospectively monitoring 10 cytokines and four growth factors in HNSCC during curative treatment [21]. The group observed a significant increase of IL-1β, IL-6, and IL-10 after completion of (chemo-)radiation. However, in the current investigation, a different cytokine and immune mediator panel, additional sampling time points during treatment and during follow-up and the impact of HPV-status were studied. Of note, 12/13 patients with oropharyngeal primaries in the previous study were HPV-positive and the majority of patients (17/30) received non-surgical treatment.

Perforin plays a pivotal role in granzyme delivery to target cells, thereby promoting cytotoxic T-cell and natural killer cell cytotoxicity [23]. Additionally, Tregs can perform autologous killing of CD4+ and CD8+ T-cells in a perforin-dependent manner [24]. Using a comprehensive cytokine and immune mediator panel, we observed a significant decrease in serum perforin levels towards the end of radiotherapy (time point 5) lasting up to 6 months after the end of treatment (time point 8) when compared to individual baseline levels (Figure 2A). Interestingly, the decrease of serum perforin concentrations during radiotherapy was accompanied by a decrease of CD8+ and CD4+ T cells during radiotherapy reaching a minimum level after the end of radiotherapy (data not shown). In vitro, chemoradiation has been reported to enhance perforin mediated tumor cell killing [25] Perforin is integrated into the cell membrane forming pores through which granzymes can enter the target cell resulting in cell killing [26]. The majority of patients received surgery with curative intent before adjuvant (chemo)radiotherapy. Thus, the tumor and the majority of the tumor immune microenvironment have been removed surgically prior to the beginning of radiation. The decreasing perforin levels may indicate that antigen-specific immune cells in the irradiated tumor microenvironment are decimated even further during radiotherapy resulting in a reduced release of perforin into the serum. This hypothesis would need further confirmation by direct assessment of the tumor microenvironment. A conflicting explanation is also possible. The prior removal of the tumor and its tumor-specific antigens reduces the number of target cells to be attacked by T cells and NK cells voiding the need for perforin release. In a setting of primary (chemo-)radiation and fractional tumor decay different results may be possible as indicated by the study of Astradsson et al. [21] and the UMAP clustering of primary CRT patients with the HPV-positive patient group in our dataset. In our study only two of the patients treated with primary CRT had sufficient consecutive samples which were available for analysis.

Interestingly, sFas (FasR) serum concentration levels increased significantly throughout the course of (C)RT starting from week 2 and lasting until the completion of radiotherapy (time point 6). sFas is the soluble form of a death receptor on the cell surface, which is responsible for apoptosis activation upon binding of its ligand (sFasL) [27]. In vitro, Fas-mediated cell killing seems not to be responsible for immune-mediated cytotoxicity after chemoradiotherapy [25]. In patients, the increasing level of soluble Fas may compete with the membranous Fas for ligand binding, resulting in reduced apoptotic stimuli to the tumor cell. In conclusion, the combination of the decrease of perforin levels and the increase of sFas concentrations point to an impaired antigen-specific immune response as a result of adjuvant(chemo)radiotherapy.

HPV-driven HNSCC has previously been shown to have a more inflamed phenotype than HPV-negative HNSCC [28]. We observed a different response to radiotherapy with regard to peripheral serum cytokine and immune mediator profiles by HPV-status. Granzyme B and MIP-1β (CCL4) showed significantly increased median serum levels during therapy in the HPV-positive group when compared to HPV-negative patients. MIP-1β (CCL4) showed a statistically significant increase from week 2 of radiotherapy until the completion of treatment (time point 6). Previous studies showed that MIP-1β is a chemoattractant, and its high intratumoral expression attracts CD8+ T-cells and NK cells [29]. High MIP-1β expression is associated with a favorable prognosis [30]. Higher levels of MIP-1β in HPV-positive patients, especially during therapy, suggest an “immune-hot” phenotype of tumors due to higher CD8+ T-cell immune infiltration. In fact, HPV-positive patients tended to have increased levels of most pro-inflammatory cytokines measured, although for the majority of cytokines these trends were not statistically significant. This may be a result of the relatively small patient sample size and the large interindividual variation of measured values. The study design did not stratify for HPV-status. Therefore, patient HPV-status is not balanced in the cohort. On the other hand, the prospective longitudinal design of the study in combination with the comprehensive cytokine and immune mediator panel measured results in a large multidimensional dataset. A Pearson cross-correlation of the seventeen serum markers from all time points was performed. In the independent correlation matrices for HPV-negative and HPV-positive patients, obvious differences between HPV-negative and HPV-positive cytokine and immune mediator profiles became evident. In HPV-positive patients, a high correlation between serum levels of all different cytokines and immune mediators was found in contrast to the HPV-negative patients, where the correlation was much lower.

To address the multidimensionality of the dataset and the complex relationships between the different cytokines and immune mediator on a level of higher abstraction, uniform manifold approximation and projection (UMAP) was performed. UMAP clustering showed that HPV status, and to a lesser extent primary therapy, seem to affect the cytokine and immune mediator expression strongly. This finding underlines that HPV-positive tumors show an inflamed phenotype characterized by dense immune cell infiltrates [10,11,31,32]. This seems to be reflected in the peripheral cytokine and immune mediator serum levels. But not only HPV-positive patients have an inflamed phenotype. The so-called IFN-γ signature, consisting of *IFNG*, *STAT1*, *CCR5, CXCL9*, *CXCL10*, *CXCL11*, *IDO1*, *PRF1*, *GZMA*, and MHCII *HLA-DRA* measured on mRNA level has been shown to predict the outcome of patients to PD-1 antibodies in HNSCC and melanoma [14]. In HNSCC treated with PD-1 antibodies the response rates and outcomes of patients are not clearly determined by HPV status: Whereas a small benefit for HPV-positive patients has been shown in Checkmate-141, this was not the case in Keynote-040 [33,34]. Our results using the 18 gene panel in the TCGA dataset which we analyzed in the serum showed a clustering of highly inflamed tumors consisting of HPV-negative and HPV-positive tumors and different primary tumor sites. Although a higher fraction of patients shows an inflamed phenotype in the HPV-positive group, in the HPV-negative group equally inflamed tumors are also present.

In the HNSCC cases with recurrent disease, we made an interesting observation. Although related samples tests between the baseline and the recurrence time point did not reveal significant differences, plasma levels of HMGB1, and serum levels of IL-4 or IL-10 were elevated around the time of recurrence. HMGB1 is a damage-associated protein acting as a chemoattractant for immune cells and has been shown to promote Treg functions [35]. HMGB1 is associated with a poor prognosis [36]. IL-4, IL-10, and IL-13 are known for their anti-inflammatory function, through the inhibition of inflammatory cytokine production [37,38]. We found an increase of HMGB1 in 4 out of 5 patients at the time point of, or shortly before the recurrence. Patients with a recurrence showed either a high overall IL-4 level or an increase in the number of inhibitory interleukins shortly before the recurrence has been observed. These findings indicate that cancer recurrence may have been accompanied by immune evasion of the tumor through HMBG1 secretion and the release of inhibitory cytokines from immune cells in the microenvironment. In line with this hypothesis, the group of Astradsson et al. observed significantly higher levels of IL-1β, IL-6, IL-8, and IL10 at week 7 of treatment in patients with recurrent disease [21].

There are certain limitations to the study. Many of the markers we assessed showed a high interindividual variance in the measured values as shown in Figure S1. For some markers, low values were measured in the majority of patients whereas others had values in magnitudes higher. Patient recruitment was limited to 18 months and within this timeframe, only 22 patients were recruited. A major factor why patients did not take part in the study was the high number of blood samples during treatment resulting in additional visits. This also resulted in missing samples from the participating patients. Imbalances in group sizes (surgical vs. non-surgical therapy, HPV− vs. HPV+) in combination with the limited cohort size and the resulting interindividual variance may have limited our findings. The serum half-lives of the cytokines and immune mediators differ immensely and are prone to treatment unspecific changes e.g., mild infections. Besides that, methodological aspects, such as sample handling, processing, and storage, also affect cytokine and immune mediator degradation and detection [39]. That is why all samples were processed and aliquoted within 1 h of sampling and only thawed once immediately before use. Measuring cytokines and immune mediators in the periphery is not directly indicative of the local situation in the tumor microenvironment, but local measurements are not possible longitudinally without repeated invasive procedures. That is why sampling from the periphery must be considered an approximation to the status in the tumor microenvironment. Many of the functions of the analyzed cytokines and immune mediators are concentration-dependent, and all samples were obtained from peripheral blood. On the other hand, this is a prospective study, monitoring patients closely throughout treatment resulting in multiple samples per patient and enabling multidimensional analysis.

## 4. Materials and Methods

### 4.1. Study Ethics

This study was performed as part of the IRECT study (Immune Response Evaluation to curative Conventional Therapy, NCT03053661). The acquisition of blood samples and clinical data was approved by the Ulm University ethics committee on 30 July 2013 (222/13) and all patients provided informed consent.

### 4.2. Patients

In this prospective, non-interventional, longitudinal study 22 newly diagnosed, histologically confirmed HNSCC patients between August 2013 and April 2015, were treated in a curative setting with either primary surgical therapy with risk-adapted adjuvant (chemo)radiotherapy (RT) or definitive chemoradiotherapy (CRT) (Arm A: RT, Arm B: CRT, Figure 1). The recommendation for first-line therapy was based on the interdisciplinary tumor board for head and neck cancer at the Comprehensive Cancer Center Ulm according to international oncologic guidelines. An overview of patient characteristics is presented in Table 1. In order to obtain a comprehensive overview of serum cytokine levels during treatment, blood samples from the 22 HNSCC patients were taken at 10 (Arm A) or 11 (Arm B) defined time points before, during and every 3 months after completion of curative treatment until 12 months (Figure 1).

### 4.3. TCGA Data and Analysis

The TCGA RNA-Seq dataset of HNSCC was downloaded from http://xena.ucsc.edu/ on 5 February 2020. The values used were log2(tpm + 0.001). Clinical parameters were derived from TCGA annotations. Hypopharynx, lip, and undetermined cancers were excluded due to low sample numbers. HPV status was based on the alignment of RNA to the HPV genome as previously defined (>1000 HPV E6/E7 RNA reads) in 279 patients [40]. For the remaining oropharyngeal HNSCC, p16 immunohistochemistry or HPV in-situ hybridization was used for the classification as HPV-positive. Patients with lacking data to determine HPV-status were excluded. In total, data from 491 patients were available.

### 4.4. Collection of Serum

Whole blood (15 mL) was collected in serum tubes (Sarstedt S-Monovette^®^ 7.5 mL) or 10 mL citrate tubes (S-Monovette^®^ 10 mL 9NC) and centrifuged for 10 min at 2500 rpm within 1 h after sampling. The supernatant was aliquoted and stored at −20 °C until use.

### 4.5. Cytokine and Immune Mediator Analysis

Serum concentrations of 17 different cytokines and immune mediators (Perforin, Granzyme A, Granzyme B, IFN-γ, GMCSF, sFas, sFasL, TNF-α, sCD137, MIP-1α (CCL3), MIP-1β (CCL4), IL-2, IL-4, IL-5, IL-6, IL-10, IL-13) were measured using a multiplex bead array assay from Merck Millipore (Milliplex Map Kit, #HCD8MAG–15 K, Burlington, MA, USA) according to manufacturer’s instructions.

Samples were measured in duplicate on a Luminex LX100 platform (Luminex Corporation Austin, TX, USA). All samples of the same patient were measured on the same plate. The data obtained were collected and analyzed via Bioplex 4.1.1 software (Bio-Rad Laboratories Inc., Hercules, CA, USA). Five-parameter logistic regression (5PL) was used to approximate the standard curves.

### 4.6. HMGB1 Detection

For the detection of HMGB1 plasma samples were used in an ELISA Kit (IBL international, #ST51011, Hamburg, Germany). The test was performed according to the manufacturer’s instructions. Optical density was measured in a TECAN infinite M200 Pro photometer at a wavelength of 450 nm.

### 4.7. Statistical and Bioinformatical Analysis

Statistical analysis and data graphing were carried out with IBM SPSS Statistics (v. 26.0), GraphPad Prism (v. 8.4.3), and R (v. 4.0.0). Comparisons between time points were analyzed using Wilcoxon signed-rank test for related samples, and with Mann–Whitney U-test for independent samples. Multiple testing correction was performed using the method of Benjamini, Krieger, and Yekutieli [41] with *q* = 0.1. Pearson correlation analysis was performed in SPSS, and the visualization in R, using the package pheatmap (1.0.12). UMAP (Uniform Manifold Approximation and Projection) [42] clustering was performed using the package uwot (0.1.8), with the default parameters (except: n_neighbours = 10, and min_dist = 0.1). UMAP visualization was done with ggplot2 (3.3.2) and ggrepel (0.8.2).

## 5. Conclusions

Our findings suggest treatment-associated changes in the concentration of a T-cell related cytokine and immune mediator panel primarily for Perforin and sFas during radiotherapy potentially impairing immune cell functions. Additionally, in HPV-positive patients, higher levels of pro-inflammatory cytokines were measured in the serum during treatment. Our findings were validated in an independent dataset (TCGA) using RNA expression data. We found indications that HMGB1 or IL-4 could be used as relapse markers during patient monitoring, but further study is needed. However, the routine monitoring of peripheral cytokines and immune mediators seems not to be warranted due to unspecific effects and low specificity for the detection of recurrence.

## Figures and Tables

**Table 1:** Patient characteristics.

Characteristics			*n*	Percentage
Primary Site	Oral cavity		5	23%
	Oropharynx		12	55%
	Hypopharynx		3	14%
	Larynx		2	9%
	Total		22	100%
Sex	Male		19	86%
	Female		3	14%
Age	Mean (range)		62.4 (50–79)	n.a.
Smoking	Pack years: median (range)		40 (0–80)	n.a.
HPV-Status (DNA/p16)	Negative		14	64%
	Positive		7	32%
	Missing		1	5%
Treatment	Primary surgery + risk-adapted adjuvant RT (Arm A)	Surgery only *	5	23%
		Surgery + adj. RT	8	36%
		Surgery + adj. CRT	5	23%
		Total Arm A	18	82%
	Primary CRT (Arm B)		4	18%
Serum Samples	*n* of serum samples per protocol		238	100%
	*n* of serum samples available		192	81%
	Patients with ≥ 8 samples		15	68%
	Patients with < 8 samples		7	32%

HPV = human papillomavirus, RT = radiotherapy, CRT = chemoradiotherapy, n.a. = not applicable. * Three patients had no indication for adj. RT based on pTNM. Two declined adjuvant RT.

## Supplementary Materials

Supplementary materials can be found at https://www.mdpi.com/1422-0067/21/17/5990/s1. Table S1. Cytokines and immune mediators overview. Figure S1. Medians of all cytokines and HMGB1 datapoints. Medians are displayed with the respective interquartile range of all available datapoints for the respective marker (all patients, all time points). The x-axis is separated into two sections and values are shown in pg/mL the x-axis. The dotted line is placed at 200 pg/mL indicating a narrow range of datapoints. Figure S2. Correlation matrix of all cytokines at all time points for the whole cohort. Pearson correlation coefficients between all cytokines and time points were graphed and hierarchical clustering was performed using Euclidian distance. Red represents a positive and blue a negative correlation. Cytokines were attributed to three groups: Purple = immune stimulatory cytokines, turquoise = immune inhibitory cytokines, green = pro-apoptotic immune mediators. Figure S3. Individual dynamics of inhibitory cytokines and HMGB1 in patients with recurrent disease. Values of (A) HMGB1 and (B) inhibitory cytokines IL-4, IL-10 and IL-13 are displayed longitudinally for five patients with recurrent disease. The time of recurrence is indicated by an arrow.

## Abbreviations

CRT/RT	(chemo-)radiotherapy
HNSCC	head and neck squamous cell carcinoma
HPV	human papillomaviruses
AJCC	American Joint Committee on Cancer
IFN-γ	interferon gamma
sFasR	soluble Fas receptor
sFasL	soluble Fas ligand
IL	interleukin
GMCSF	granulocyte-macrophage colony-stimulating factor
TNFα	tumor necrosis factor alpha
MIP	macrophage inflammatory proteins
HMGB1	high mobility group box 1
UMAP	Uniform Manifold Approximation and Projection
NK cells	natural killer cells
TCGA	The Cancer Genome Atlas
ELISA	enzyme-linked immunosorbent assay
