# Supplementary material for: Peripheral Cytokine Levels Differ by HPV Status and Change Treatment-Dependently in Patients with Head and Neck Squamous Cell Carcinoma
Source: Int J Mol Sci. 2020 Aug 20;21(17):5990. doi: 10.3390/ijms21175990 (PMC7503943; doi:10.3390/ijms21175990)
Supplement: Supplementary file 1 [file ijms-21-05990-s001.pdf]

Supplementary Figure Legends

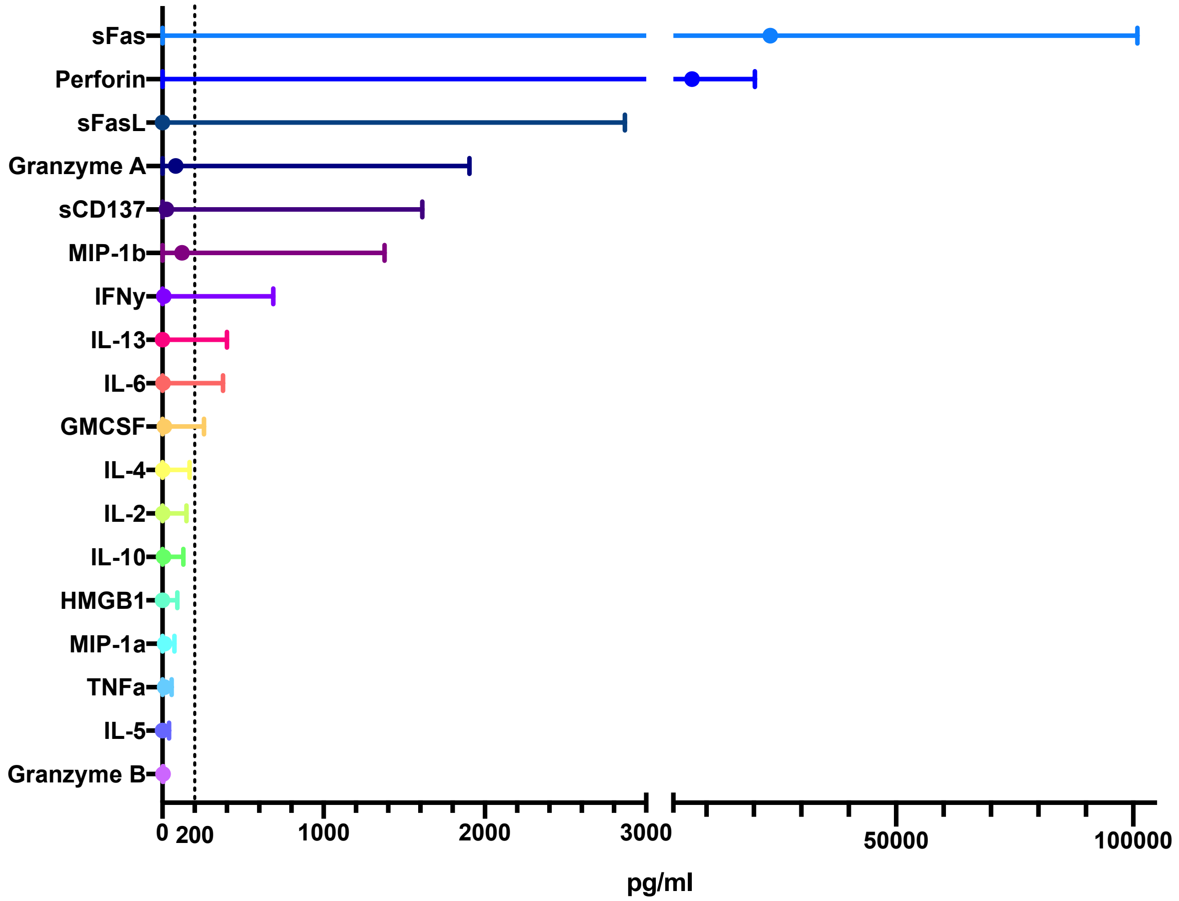

Supplementary Figure S1: Median and IQR of all datapoints per cytokine
Median and interquartile range of all datapoints (all patients, all timepoints) are displayed for each cytokine / immune mediator. The values in pg/ml are shown on the x-axis.

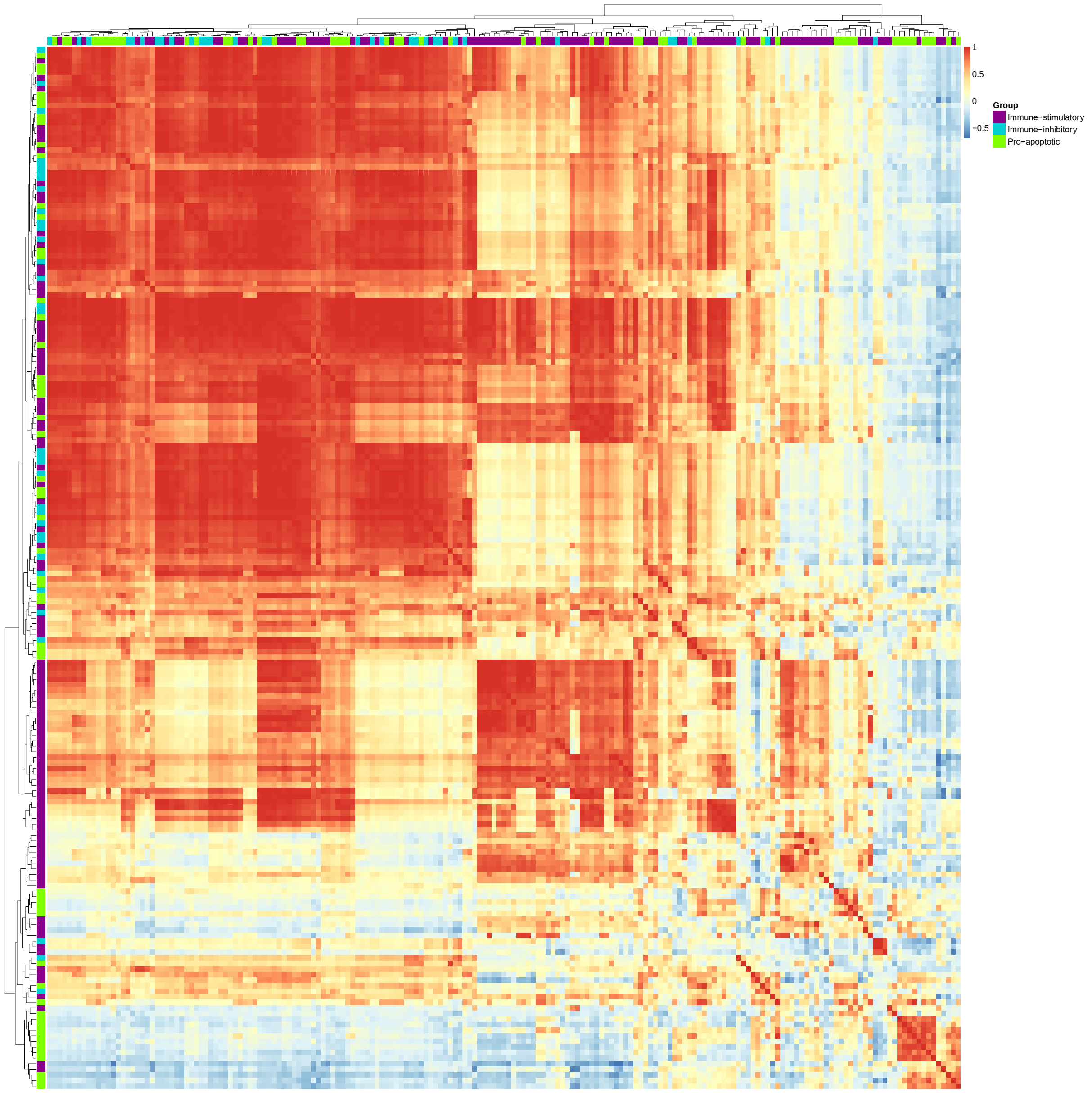

Supplementary Figure S2: Heatmap of correlation matrix.

Correlation matrix of all cytokines and immune mediators at all time points. Pearson correlation coefficients between all cytokines and time points were graphed and hierarchical clustering was performed using Euclidian distance. Red represents positive, and blue negative correlation. Cytokines were attributed to three groups: Purple = immune stimulatory cytokines, turquoise = immune inhibitory cytokines, green = pro-apoptotic immune mediators.

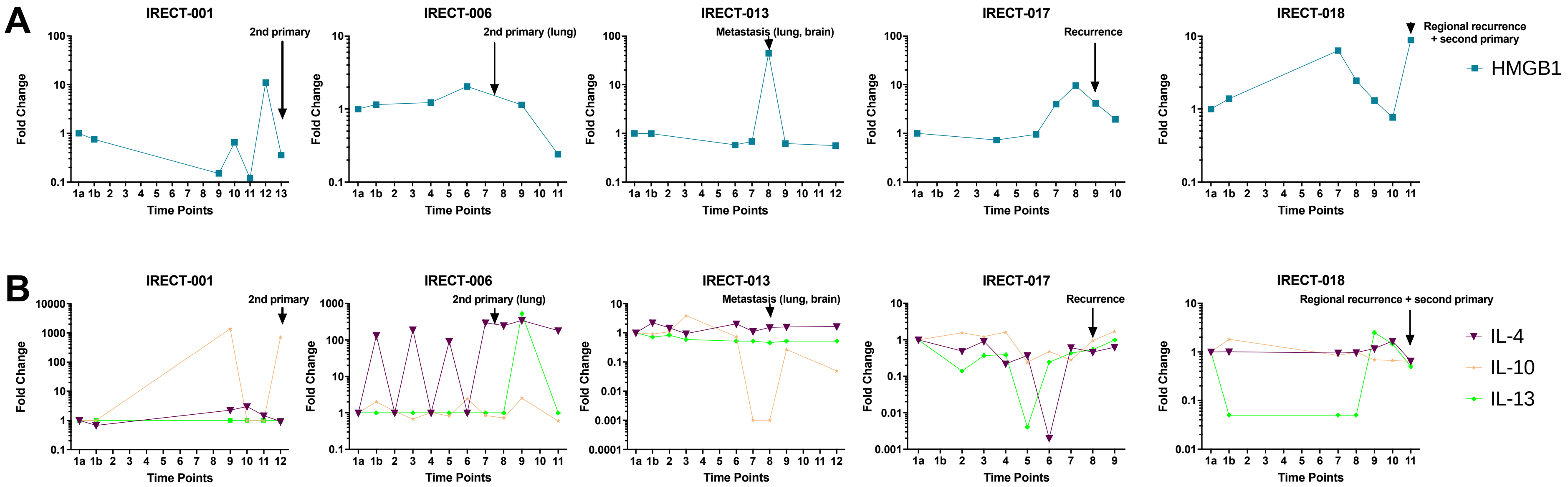

Supplementary Figure S3: HMGB1 and inhibitory cytokines in relapsed patients.

HMGB1, IL-4, IL-10 and IL-13 values were graphed longitudinally for patients with disease relapse. The time of recurrence is indicated with an arrow. The x-axis shows the timeline, the y axis shows the fold change normalized to the individual patient's baseline.
